# Patients' functioning as predictor of nursing workload in acute hospital units providing rehabilitation care: a multi-centre cohort study

**DOI:** 10.1186/1472-6963-10-295

**Published:** 2010-10-29

**Authors:** Martin Mueller, Stefanie Lohmann, Ralf Strobl, Christine Boldt, Eva Grill

**Affiliations:** 1 Institute for Health and Rehabilitation Sciences (IHRS), Ludwig-Maximilians-Universität, Munich, Germany; 2 ICF Research Branch of WHO FIC CC (DIMDI) at SPF Nottwil, Nottwil, Switzerland, and at IHRS, Ludwig-Maximilians-Universität, Munich, Germany; 3 Swiss Paraplegic Research, Nottwil, Switzerland

## Abstract

**Background:**

Management decisions regarding quality and quantity of nurse staffing have important consequences for hospital budgets. Furthermore, these management decisions must address the nursing care requirements of the particular patients within an organizational unit. In order to determine optimal nurse staffing needs, the extent of nursing workload must first be known. Nursing workload is largely a function of the composite of the patients' individual health status, particularly with respect to functioning status, individual need for nursing care, and severity of symptoms. The International Classification of Functioning, Disability and Health (ICF) and the derived subsets, the so-called ICF Core Sets, are a standardized approach to describe patients' functioning status. The objectives of this study were to (1) examine the association between patients' functioning, as encoded by categories of the Acute ICF Core Sets, and nursing workload in patients in the acute care situation, (2) compare the variance in nursing workload explained by the ICF Core Set categories and with the Barthel Index, and (3) validate the Acute ICF Core Sets by their ability to predict nursing workload.

**Methods:**

Patients' functioning at admission was assessed using the respective Acute ICF Core Set and the Barthel Index, whereas nursing workload data was collected using an established instrument. Associations between dependent and independent variables were modelled using linear regression. Variable selection was carried out using penalized regression.

**Results:**

In patients with neurological and cardiopulmonary conditions, selected ICF categories and the Barthel Index Score explained the same variance in nursing workload (44% in neurological conditions, 35% in cardiopulmonary conditions), whereas ICF was slightly superior to Barthel Index Score for musculoskeletal conditions (20% versus 16%).

**Conclusions:**

A substantial fraction of the variance in nursing workload in patients with rehabilitation needs in the acute hospital could be predicted by selected categories of the Acute ICF Core Sets, or by the Barthel Index score. Incorporating ICF Core Set-based data in nursing management decisions, particularly staffing decisions, may be beneficial.

## Background

Nurses play a major role in the acute care hospital by improving or maintaining the health status and functioning of patients, while minimizing their distress and suffering [1]. Since nurse staffing represents a considerable proportion of the total staffing costs in acute care hospitals [2], management decisions regarding quality and quantity of nurse staffing have important consequences for hospital budgets. The task of management is to obtain an optimal solution to the nursing care requirements of particular patient populations within an organizational unit. The cost-benefit analysis implicit in the process is essential because nurse staffing is closely associated with patient outcomes [3-5]. Furthermore, sufficient nurse staffing to avoid excessive workload is an essential requirement for occupational health of caregivers [6].

Various criteria are available to determine whether the scale and composition of nurse staffing are adequate to meeting the needs of patients and staff [7]. In general, these criteria include factors such as nurses' educational level, ward size or number of beds, acute versus chronic status of patients, and the time required for individual nursing interventions. Regression analysis has sometimes been used to account for the most important determinants, e.g. bed occupancy. Various methods are available for quantifying the nursing workload, which is arguably one of the most important management tools to optimize staffing decisions. Irrespective of the method employed for calculating staffing requirements and for accurate prediction of the consequent workload, the relevant factors determining workload must first be determined [8].

Nursing workload is largely determined by patients' individual health status, which is the composite of patients' functioning status, the individual need for nursing care, and the severity of symptoms [8]. Research on nursing-related workload has typically been limited to consideration of the day-to-day work activities of nurses and the amount of time spent for each activity [9-11]. The occurrence of specific association of nursing workload with patients' functioning seems self-evident, but the ramifications of this relationship have not hitherto been explored. It is known that the extent of a patient's functional dependence in basic activities of daily living (ADL), which can be measured by the Barthel Index [12] or other instruments, is a predictor of increased nursing workload [13,14]. However, this approach may be incomplete with respect to the current understanding that nurses not only compensate or relieve disability, but also serve in a positive manner to optimize their patients' functioning and health [15]. In addition, many instruments like the Barthel Index use summary scores, which summate the scores of single items, so as to describe the extent of functioning and disability. Such summation scores are known to mask the influence of particular aspects of functioning and disability reflected by particular Barthel Index items. This masking may lead to imperfect assessment and conclusions, which would decrease the usefulness of the Barthel Index for clinical practice [16]. Furthermore, the Barthel Index tends to reduce the complex picture of human functioning to a consideration only of motoric aspects of self-care, without consideration of the relevant cognitive aspects [17,18]. Therefore, it is of great practical interest to explore which aspects of functioning and disability are drivers for nursing workload, with particular consideration of aspects scarcely reflected in ADL scores, such as impairments in body functions and structures, or modifying contextual factors. The International Classification of Functioning, Disability and Health (ICF) is a potentially interesting option for describing single components of functioning and specific goals of nursing care. Introduced by the World Health Organization to classify and structure human functioning in all its facets, the ICF is specifically intended for use by all types of health professionals for the standardized documentation of patients' health [19]. The ICF approach is generally applicable, regardless of the underlying health condition or clinical situation, and is comprehensive across diverse elements of functioning. While the entire ICF is too extensive for routine use, the abridged versions, called ICF Core Sets, are designed for application to specific health conditions or settings, such as the acute care hospital [20]. We have recently shown that certain distinct categories of the ICF, which describe relevant aspects of functioning in the acute situation, also correspond to goals of nursing interventions [21]. The occurrence of this relationship shows that patients' functioning, as described most especially in the ICF Core Sets, is indeed highly relevant for nursing care. It follows that patients' functioning as expressed by ICF categories is likely to be inherently associated with nursing workload. Nonetheless, the ICF categories have not hitherto been tested as predictors of nursing workload. Moreover, the implementation of the ICF in clinical applications - assessment and outcome evaluation - by developing ICF Core Sets is a major endeavour of WHO [22]. Therefore it should be explored, to what extent ICF Core Set data is useful in further applications, such as predicting resource utilization.

Based on these considerations, the objective of the present study was to examine the associations between patients' functioning, expressed with ICF Core Set categories, and nursing workload for patients in the acute care situation. Specifically, we aimed to

(1) examine which aspects of functioning as described by the Acute ICF Core Sets were the best predictors of nursing workload.

(2) compare the amounts of variation of nursing workload explained by the Acute ICF Core Sets and the Barthel Index, and

(3) validate the Acute ICF Core Sets in terms of their ability to predict nursing workload.

## Methods

### Study design, setting and participants

We conducted a multi-centre cohort study on patients undergoing rehabilitation in the acute hospital. The available data were derived from a larger study conducted for the development and validation of ICF Core Sets in the acute and early post-acute setting, which is not yet reported. Patients were recruited consecutively between May 2005 and August 2007 from three university-affiliated acute hospitals: the Kaiser-Franz-Joseph-Spital Vienna (Austria) (KSFS), the University Hospital Zurich (Switzerland) (USZ), and the University Hospital Heidelberg (UHH) (Germany). Patients were included if they were at least 18 years old and received coordinated rehabilitation interventions for treatment of any acute musculoskeletal, neurological or cardiopulmonary injury or disease [23]. Informed consent was obtained from patients, or, if the patient was unable to make an informed decision, from the patient's caregiver. Approval of the study was obtained from the institutional ethics committees from all involved hospitals prior to start (Ethics committee of the City of Vienna: *EK 05-087-0805*, Ethics commission of the Canton of Zurich: *ref. 569*, Ethics commission of the Medical Faculty of Heidelberg: *ref. 096/2005*).

### Variables

#### Nursing workload

Nursing workload was documented using an instrument for the documentation of nursing activities known as LEP ("Leistungserfassung in der Pflege"). LEP was developed in Switzerland as a nursing workload classification for documenting daily nursing activities, and has come to be widely used in German-speaking countries because of the simplicity of its application to clinical practice [24]. The main part of LEP consists of the "Nursing Interventions Catalogue" which contains 15 chapters of nursing areas (e.g. movement, personal hygiene/dressing or eating/drinking). These chapters include a total of 79 different nursing interventions, which are stratified into four levels of complexity: very simple, basic, complex, and very complex. In total, the LEP provides 163 different nursing intervention variables [25]. They are structured by name, description, remarks, instructions, and an assigned time expenditure [26]. In the present study, we used a selection of 32 nursing interventions with a total of 84 LEP nursing intervention variables, which we have previously shown to be of particular relevance in the acute and early post-acute situation [21] (see table 1).

**Table 1:** LEP nursing interventions relevant for the acute and post-acute situation resulting in a total of 84 items*

Activity and Recreation	Bed Preparation
Cardiac support	Case conference
Compressions	Drainage/Irrigation
Eating/Drinking	Elimination
Escort	Extubation
Infusion	Inhalation
Inserting catheter/tube	Intubation
Isolation procedures	Massage
Mobilising	Nursing Visit
Obtaining and fitting support aids	Occupational Therapy
Oral/nasal/tracheal suctioning	Oxygen therapy
Patient-nurse communication/information-giving	Perceptual training
Personal Hygiene/Dressing	Physician Support
Positioning	Respiratory support
Technical Procedure	Therapeutic Intervention
Tube change	Wound Dressing/Wound Care

*LEP nursing interventions are detailed up to four specifications, e.g. 'Mobilising very simple' - 'Mobilising basic' - 'Mobilising complex' - 'Mobilising very complex'.

Detailed day-to-day nursing workload data, expressed in units of expended minutes per day, was available from one study centre, the USZ. In the other two study centres, nursing workload data was collected during the first and the last 24 hours of hospital stay. To estimate the total nursing workload for all study centres by a common formula, we estimated the total workload using the approximation

(I) $$\frac{{\text{nursing workload}}_{\text{first day}}+{\text{nursing workload}}_{\text{lastday}}}{2}\cdot \text{length of stay}.$$

We explored the robustness of this approximation on the known total nursing workload of the USZ. The goal of this preliminary analysis was to find the best model based on the first and last day of stay to predict workload. Results presented in Table 2 showed that the method in (I) gave a practical and parsimonious estimation of nursing workload, explaining over 80% of the variation of workload with an intercept and over 90% without an intercept. It is valid to calculate without an intercept, as the regression line should in theory cross the zero-point.

**Table 2:** Summary of considered formulas to estimate total nursing workload: A higher coefficient indicates that the estimate corresponds more closely to the actual sum of workload.

Model	with intercept		without intercept	
	Coef.	Rsquared	Coef.	Rsquared
I) total LEP ^a ^~ LEP (Day 1) * LOS ^b^	0,56	0,75	0,62	0,85
II) total LEP ~ LEP (Last Day) * LOS	0,93	0,38	1,2	0,61
III) total LEP ~ Mean(LEP(Day 1, Last Day)) * LOS	0,93	0,82	0,96	0,9

Results were based on the data available at the USZ. Total LEP was calculated as the sum of the daily LEP minutes.

^a ^LEP = nursing workload in LEP minutes, ^b ^LOS = Length of stay

#### ICF Core Sets

Patients' functioning was assessed at admission using the Acute ICF Core Sets. As noted above, the Acute ICF Core Sets are sets of categories of the International Classification of Functioning, Disability and Health (ICF), which are of empirically proven relevance for patients with musculoskeletal, cardiopulmonary or neurological conditions in the acute hospital [20]. The ICF was introduced by the World Health Association (WHO) in 2001 to provide a comprehensive framework of functioning, health, and health-related domains. The WHO intended the ICF to facilitate communication between different users, in particular health care workers, researchers, and policy makers, as well as the general public. Since the ICF organizes in a standardized manner all domains of functioning and contextual factors encountered in human life, it can be regarded as the prototypical framework in health care. This framework encompasses the frequency, distribution, determinants and consequences of functioning in relation to health conditions, and also considers personal and environmental factors. The ICF has two parts: Part one covers the components body functions (b), body structures (s) and activities and participation (d), whereas part two covers contextual factors including the components environmental factors (e) and personal factors. The ICF is a systematic classification, in which the letters (b, s, d and e) refer to the specified components of the classification. The letters are followed by a numeric code starting with the chapter number (one digit), which is followed by the second level (two digits), and the third and fourth levels (one digit each).

The entire ICF contains 1424 categories, making it too comprehensive to serve as a day-to-day tool in clinical practice, or in population-based studies. Therefore, it has been adapted or abridged according to the perspectives and needs of different users. Indeed, this adaptation was the primary motivation for the development of ICF Core Sets, which constitute minimal and sufficient standards for the assessment and reporting of functioning, disability and health.

As noted above, ICF core sets have been developed for three main indications, i.e. neurological, musculoskeletal, and cardiopulmonary conditions. The ICF Core Set for patients with neurological conditions in the acute hospital consists of 85 second-level ICF categories (41 of the component Body Functions, five from the component Body Structures, 18 from the component Activities and Participation and 21 from the component Environmental Factors) [27]. The ICF Core Set for patients with musculoskeletal conditions in the acute hospital consists of 47 second-level ICF categories (17 of the component Body Functions, nine from the component Body Structures, 11 from the component Activities and Participation and 10 from the component Environmental Factors) [28]. Finally, the ICF Core Set for patients with cardiopulmonary conditions in the acute hospital consists of 48 second-level ICF categories (21 of the component Body Functions, four from the component Body Structures, 10 from the component Activities and Participation and 13 from the component Environmental Factors) [29].

For each of these categories, the ICF provides qualifiers which range from 0 (no impairment/limitation) to 4 (complete impairment/limitation) to quantify functioning and disability by rating the severity of the problem in the different ICF categories. Because the metric properties of all qualifiers are not yet evaluated sufficiently, we use for the present only three qualifiers instead of five. Each category of the components Body Functions and Activities and Participation was graded with the qualifiers 0 for "no impairment/limitation", 1 for "moderate impairment/limitation" and 2 for "severe impairment/limitation". The categories of the component Body Structures were graded with the qualifiers 0 for "no impairment" and 1 for "impairment". The categories of the component Environmental Factors were graded with 0 for "no barrier/facilitator" and 1 for "barrier/facilitator".

#### Barthel Index

In addition to ICF assessment, we also assessed patients' functioning at admission using the Barthel Index (BI), which is a 10-item instrument measuring disability in terms of the level of functional independence obtainable by a person in personal activities of daily living (ADL) [12,30,31]. The BI has been used for four decades for the assessment of patients through direct observation and review of medical records by trained health professionals. Of the 10 ADLs, eight can be described as self-care activities and two as mobility-related activities [32]. The scores assigned to each item are based on the time and amount of actual physical assistance required, in cases when a patient is unable to perform the activity. The BI weights each of the ten functions separately, giving a final score that ranges from 0 (totally dependent) to 100 (totally independent) [12]. Due to its reliability and validity, the BI has been proposed as a standard measure for disability and has recently come to be used in the context of clinical nursing care for determine patients' needs in different settings [33,34].

#### Sociodemographic Variables

To account for possible confounding, age at admission and sex were recorded for every patient.

### Statistical analysis

#### Descriptive Analysis

For categorical variables, we calculated absolute and relative frequencies along with their 95% confidence intervals. For continuous variables, we calculated mean, median and standard deviation. Patient characteristics were provided for the whole sample and stratified for condition groups according the Acute ICF Core Sets (musculoskeletal, cardiopulmonary and neurological conditions).

#### Models with Barthel Index

To assess the association of the BI score at admission with the total nursing workload during hospital stay we used multiple linear regression models adjusted for age and sex. Separate analyses were carried out for each of the three indication groups.

#### Models with ICF Categories

**Figure 1:**
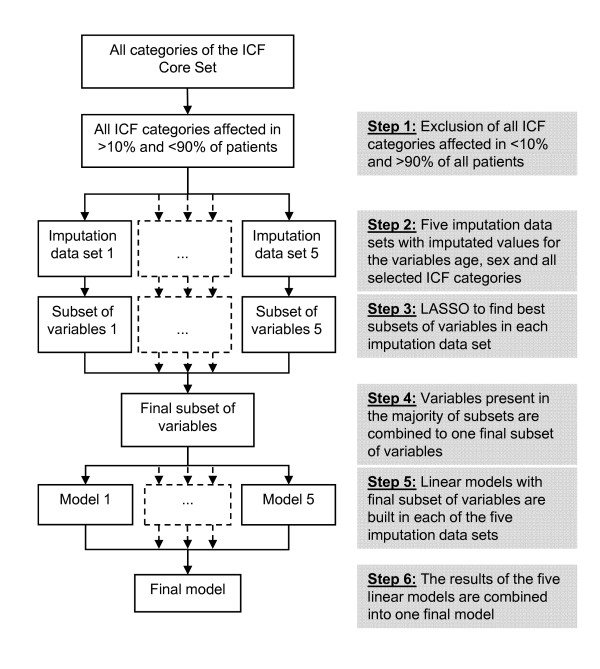
**Process of variable selection**.

To assess the association of the patients' functioning at admission described by ICF Core Sets categories with total nursing workload during hospital stay we used multiple linear regression models adjusted for age and sex. Separate analyses were carried out for each of the three indication groups. In order to find the subset of variables most suitable for predicting total nursing workload, we used a serial selection process, which includes multiple imputation of missing data. The selection process consists of six steps as follows, and as summarized in Figure 1:

Step 1: ICF categories impaired or limited in less than 10% or in more than 90% of the patients were excluded from further analyses, as they show too little variation.

Step 2: Missing values in the variables 'age' and in the ICF categories were replaced by multiple imputation, using the function 'mice' of the software package 'mice' of R 2.11.0 [35]. This procedure uses Gibbs sampling for multiple imputations for incomplete multivariate data. Multiple imputation is a useful method for reducing bias and increasing precision when data matrices are incomplete [36]. Multiple imputation has several advantages over complete-case analyses, wherein deleted observations lead to diminished precision of the outcome measure, resulting from the reduction of sample size and consequent bias, when the missing data are not randomly distributed. Simulation studies demonstrated that with five generated data sets, multiple imputations yield valid results [37,38]. Thus, we generated five data sets with differently imputated variables. All further steps of the multiple analyses were carried out with each of the five imputation data sets.

Step 3: In order to improve prediction accuracy, and to extract small subsets of independent variables seemingly with the strongest effects on the dependent variable, we used the least absolute shrinkage and selection operator (LASSO) [39] to identify which of the ICF categories are independently associated with the log-transformed workload. The LASSO minimizes the residual sum of squared errors, with a bound on the sum of the absolute values of the coefficients. To avoid large variance, which often arises from ordinary least square regression, the LASSO sets some regression coefficients to zero and shrinks others based on a preset regularization parameter, the so-called penalty. Thus, the method acts as a tool for identifying valid subsets of ICF categories. The penalty value was chosen by leave-one-out cross-validation [40]. By this process we obtained five subsets of predictor variables from each of the five imputation data sets.

Step 4: We selected a final subset of predictor variables consisting of those variables present in the majority of the five subsets of the imputation data sets.

Step 5: The final subset of variables was entered into a linear model in each of the five imputation data sets. The outcome variables of these models were total nursing workload as measured with LEP and as estimated with formula (I).

Step 6: A final model was created by averaging the coefficients of all of the five models built in the imputation data sets.

#### Comparison of the models

For each of the three indication groups, the percentage of variance (R-squared) of total nursing workload explained by the model based on the BI score was compared to the percentage of variance explained by the model based on the ICF.

All statistical analyses were performed using R 2.11.0 [41]

## Results

### Baseline characteristics

A total of 271 patients were included into the analysis. Of these, 77 (28.4%) had neurological conditions, 127 (46.9%) had musculoskeletal conditions and 67 (24.7%) had cardiopulmonary conditions. 53.9% of the patients were female (95% CI 47.7, 59.9). Patients had a mean age at admission of 67.0 years (range: 18-100, SD 17.35) and a median age of 71 years. The mean total nursing workload was 4418 minutes (range: 278-78910, SD 6827) with a median of 2200 minutes. The mean daily nursing workload was 276 minutes (range 58-1835). The mean length of stay was 14.4 days (range: 2-64, SD 12.6) with a median of 10 days. The mean BI Score at admission was 55.3 points (range: 5-100, SD 24.8) with a median of 50 points. Patient characteristics stratified for condition group are presented in table 3.

**Table 3:** Patient characteristics stratified for condition group

Variable	Neurological conditions (n = 77)	Musculoskeletal conditions (n = 127)	Cardiopulmonary conditions (n = 67)
	**Mean (range)**	**Mean (range)**	**Mean (range)**
Age (yrs)	65 (18-95)	64 (21-94)	75 (22-100)
LEP minutes	5798 (416-78910)	3943 (278-29080)	3732 (397-37260)
Length of stay (days)	18 (2-57)	14 (2-64)	11 (2-59)
Barthel Index at admission	59 (5-100)	50 (20-85)	57 (5-100)
Daily LEP time (minutes)	286 (74-1835)	245 (58-554.5)	322 (126-990)
	**Number of patients (%)**	**Number of patients (%)**	**Number of patients (%)**
Female	38 (49.4)	74 (58.3)	34 (50.7)

### Multiple analysis

Model assumptions were checked using histograms and QQ-plots. The QQ-plot revealed a right-skewed distribution of the variable 'Total nursing workload'. We performed a logarithmic transformation in order to achieve normal distribution. All other metric variables were approximately normally distributed.

#### Neurological conditions

##### Model with Barthel Index

Data from 49 patients were available for multiple analyses. The BI score, age and sex explained 47.6% of the variance of total nursing workload. Details on the model are presented in table 4.

**Table 4:** Variables predicting nursing workload in patients with neurological conditions selected by the 'least absolute shrinkage and selection operator' (LASSO).

	Model with Barthel Index n = 49		Model with ICF categories n = 77	
**Variable**	**Estimate**	**P-Value**	**Estimate**	**P-Value**
**Age**	0.01	0.4085	0	0.5137
**Sex (male)**	-0.32	0.1588	-0.35	0.0774
**Barthel Index**	-0.02	<.0001		
**b147 **Psychomotor functions			0.32	0.0517
**b525 **Defecation functions			0.65	0.0061
**b620 **Urination functions			0.22	0.0797
**d420 **Transferring oneself			0.07	0.7176
**d520 **Caring for body parts			0.41	0.0674
**R-squared (R-squared adjusted for number of variables in the model)**	0.4758 (0.4409)		0.4928 (0.4414)	

##### Model with ICF categories

In patients with neurological conditions, the ICF categories *Sleep functions *(b134) and *Haematological system functions *(b430) were excluded from the analyses because of doubts about the quality of the data collection. Data from 77 patients were available for multiple analyses. Of the 104 ICF categories then remaining, 16 categories were excluded from further analyses, by the criterion that they were impaired or restricted in either less than 10% or more than 90% of all patients with neurological conditions. Thus, 88 categories of the ICF Core Set for neurological conditions were retained for the multiple linear models.

**Figure 2:**
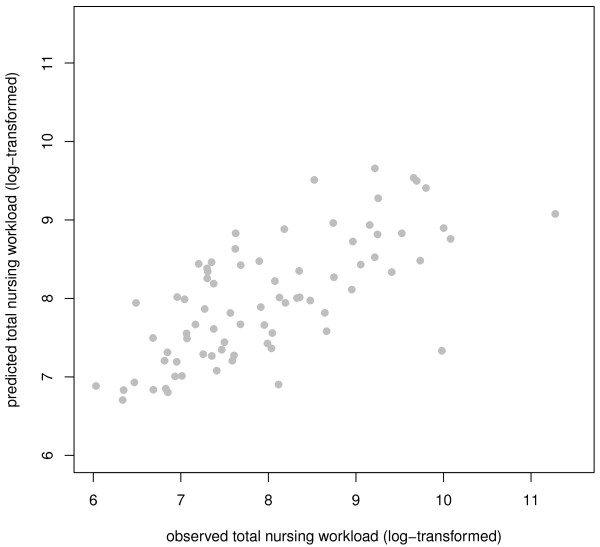
**Plot of fitted vs. observed value for the sum of the total nursing workload (patients with neurological conditions)**.

The LASSO was carried out in each of the five imputed data sets with the 88 ICF categories: 'Total nursing workload' was the outcome variable and sex and age were forced-in variables. *Defecation functions *(b525), *Psychomotor functions *(b147)*, Caring for body parts *(d520), *Urination functions *(b620) and *Transferring oneself *(d420) were identified as the subset of variables exhibiting the strongest effect on total nursing workload. These seven variables were entered into linear models in the five imputation data sets. The final model explained 49.3% of the variance of total nursing workload (see table 4 for details). Accuracy of the fit is visualized in figure 2.

#### Musculoskeletal conditions

##### Model with Barthel Index

50 patients were available for multiple analyses. BI score, age and sex explained 21.2% of the total variance of total nursing workload. Details of the model are presented in table 5.

**Table 5:** Variables predicting nursing workload in patients with musculoskeletal conditions selected by the 'least absolute shrinkage and selection operator' (LASSO).

	Model with Barthel Index n = 50		Model with ICF categories n = 127	
**Variable**	**Estimate**	**P-Value**	**Estimate**	**P-Value**
**Age**	0.01	0.4282	0.02	<.0001
**Sex (male)**	0.18	0.5244	0.33	0.0278
**Barthel Index**	-0.03	0.0029		
**b130 **Energy and drive functions			0.3	0.0291
**b620 **Urination functions			0.34	0.0018
**R-squared (R-squared adjusted for number of variables in the model)**	0.2125 (0.1611)		0.2276 (0.1957)	

##### Model with ICF categories

In patients with musculoskeletal conditions, the ICF category *Sleep function *(b134) was excluded from the analyses because of doubts about the quality of the data collection. Data from 127 patients were then available for multiple analyses. Of the remaining 56 ICF categories, 18 categories were excluded from further analyses, as they were impaired or restricted in either less than 10% or more than 90% of all musculoskeletal patients. Thus, 38 categories of the ICF Core Set for musculoskeletal conditions entered the modelling process.

**Figure 3:**
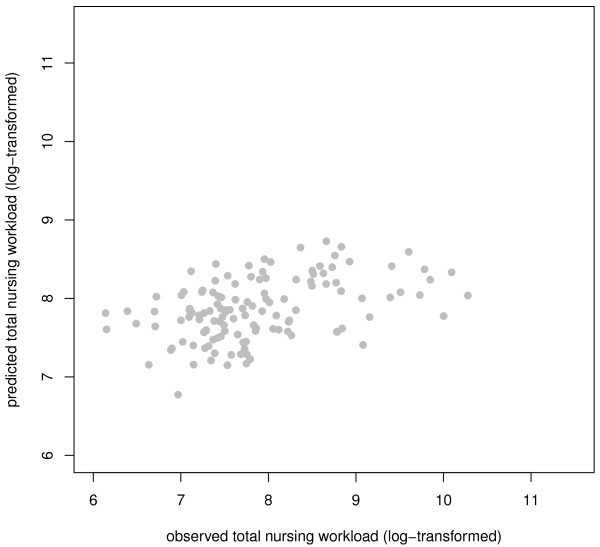
**Plot of fitted vs. observed value for the sum of the total nursing workload (patients with musculoskeletal conditions)**.

Five imputation data sets were created for patients with musculoskeletal conditions. The LASSO was carried out for each of these data sets with the 38 ICF categories: 'Total nursing workload' was the outcome variable and the variables sex and age were forced-in variables. *Energy and drive functions *(b130) and *Urination functions *(b620) were identified as the subset of variables exhibiting the strongest effect. These four variables were entered into linear models in the five imputation data sets. The final model explained 22.8% of the variance of total nursing workload (see table 5 for details). Accuracy of the fit is represented in figure 3.

#### Cardiopulmonary conditions

##### Model with Barthel Index

Fifty patients were available for multiple analyses. BI score, age and sex explained 39.2% of the total variance of Total nursing workload. Details of the model are presented in table 6.

**Table 6:** Variables predicting nursing workload in patients with cardiopulmonary conditions selected by the 'least absolute shrinkage and selection operator' (LASSO)

	Model with Barthel Index n = 50		Model with ICF categories n = 67	
**Variable**	**Estimate**	**P-Value**	**Estimate**	**P-Value**
**Age**	0.01	0.2095	0.01	0.1982
**Sex (male)**	-0.19	0.3974	-0.09	0.6691
**Barthel Index**	-0.02	<.0001		
**d410 **Changing basic body position			0.34	0.2581
**d420 **Transferring oneself			0.12	0.6965
**d530 **Toileting			0.16	0.5441
**d540 **Dressing			0.4	0.1417
**R-squared (R-squared adjusted for number of variables in the model)**	0.3918 (0.3521)		0.4137 (0.3551)	

##### Model with ICF categories

In patients with cardiopulmonary conditions, the ICF categories *Sleep functions *(b134) and *Haematological system functions *(b430) were excluded from the analyses because of doubts about the quality of the data collection. Data from 67 patients were available for multiple analyses. Of the remaining 59 ICF categories, 14 categories were excluded from further analyses, as they were impaired or restricted in either less than 10% or more than 90% of all cardiopulmonary patients. Thus, 45 categories of the ICF Core Set for cardiopulmonary conditions were entered into the modelling process.

**Figure 4:**
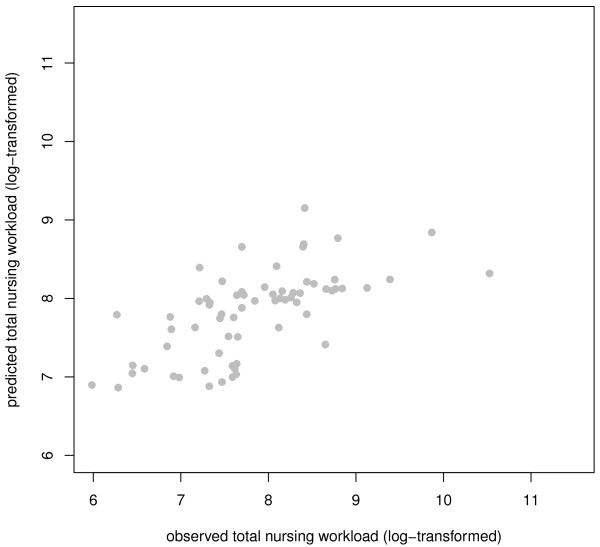
**Plot of fitted vs. observed value for the sum of the total nursing workload (patients with cardiopulmonary conditions)**.

Five imputation data sets were created for patients with cardiopulmonary conditions. The LASSO was carried out in each of these data sets with the 16 preselected ICF categories: 'Total nursing workload' was the outcome variable and sex and age were forced-in variables. The variables *Changing basic body position *(d410), *Transferring oneself *(d420), *Toileting *(d530) and *Dressing *(d540) were chosen as the subset of variables exhibiting the strongest effect on total nursing workload. These six variables were entered into linear models in the five imputation data sets. The final model explained 41.4% of the total variance of nursing workload (see table 6 for details). Accuracy of the fit is represented in figure 4.

## Discussion

The aim of the current study was to examine the factors leading to variation in the nursing workload in the acute hospital setting in order to provide a basis for better management decision-making. To our knowledge, we present the first instance of applying ICF to the task of assessing the nursing workload, an outcome highly relevant to the optimization of patient care and allocation of nursing resources. The results of our study indicate that up to one half of the variation in patient-related nursing workload observed for patients with rehabilitation needs in the acute care situation can be predicted by relatively few categories of the ICF. This held equally for patients with neurological, cardiopulmonary, and musculoskeletal conditions. We also found that the use of selected ICF Core Set categories was equally predictive as the Barthel Index, thus demonstrating the predictive validity of the ICF Core Sets.

### ICF-Models

We found that the selections of ICF categories predicting nursing workload differed according to the underlying health condition, and were in line with the current literature [42,43].

In patients with neurological conditions, the predominant diagnosis was stroke. Impairment in elimination functions (*Urination *and *Defecation functions*) and limitations in self-care (*Caring for body parts*) and limitations related to movement (*Transferring oneself*) and *Psychomotor functions *(including speed and quality of response and behaviour) had the greatest influence on nursing workload in these patients. This no doubt reflects the widespread neurological and functional consequences of stroke, which can manifest in cognitive and senso-motoric deficits leading to significant impairments in basic daily activities, such as elimination or personal hygiene, all of which are highly demanding of nursing care [44].

In patients with musculoskeletal conditions, impairments in *Urination functions *and *Energy and drive functions *appeared to have the greatest influence on nursing workload. The ICF category *Urination functions *comprises the ability to void the urinary bladder, and to control urination. Limitations in these functions increases nursing workload due to the requirements for catheterization or use of absorbent products [45]. The ICF category *Energy und drive functions *comprises the degree of motivation and energy experienced by the patients. It is known that positive personal motivation is a major factor for a successful rehabilitation process, with predictable consequences for the nursing workload [46].

In patients with cardiopulmonary conditions, the limitations in basic aspects of mobility (*Changing basic body position*, such as lying down, standing or sitting and *Transferring oneself*) and self-care (*Toileting *and *Dressing*) had the greatest influence on nursing workload. A main component of nursing care in the acute situation after a cardiopulmonary episode consists of monitoring the patient's activity level. The reasons for this are two-fold: First, patients have to be monitored closely, since most complications appear shortly after the acute event. Second, early mobilisation measures have to be carried out judiciously in order to optimize the restoration of the mobility level [47]. Accordingly, nurses in the acute situation have to support and supervise patients in most of their self-care activities.

### Comparison of ICF-Models with Barthel Index Models

When comparing the aptness of the BI score and single ICF categories for predicting nursing workload, the difference in explained variation appears marginal. However, additional factors have to be kept in mind when considering the benefits of the two approaches. In particular, this study was not intended to promote ICF as a universal competitor for the BI, which is a well-established and justified measure. Measures like the BI are routinely applied in rehabilitation facilities to monitor and estimate patient progress and nursing workload, but not previously in the acute hospital situation. In this novel context, approaches like the Acute ICF Core Sets are potentially more useful since they comprise not only the rehabilitation perspective but also the acute medical and nursing perspective [23]. Here, we find the ICF to be just as useful as the BI but more efficient in predicting variation in nursing workload in the acute situation given the small number of items in the ICF. As mentioned above, the BI contains ten single items which have to be rated for calculation of the total BI score. In patients with neurological conditions, we were able to explain more variance with five ICF categories than was possible with the BI, whereas two ICF categories likewise sufficed for patients with musculoskeletal conditions and four ICF categories for patients with cardiopulmonary conditions. As such, the ICF may emerge as the preferred approach for efficiently predicting workload in the acute hospital, given its greater flexibility, and also on the grounds of parsimony and ease of application. In this context, it is important to emphasize that the ICF Core Sets are in general intended to address the whole spectrum of patients' problems with a specific health condition and/or in a determined clinical setting [20]. Moreover, there are some situations in which health professionals have to focus on the burden of care of patients in particular patients groups. In the field of clinical management, this latter task is of major importance. Health professionals must make rapid and efficient decisions regarding the best and most patient-oriented way to allocate the available resources. The ICF categories identified in this study are a selection from the entire set of potential areas of patients' functioning. Our procedures identified the minimal subset of categories that give the best prediction of nursing workload. As such, the ICF categories identified in the present study can be considered as a brief and fast assessment, with particular relevance to the task of nursing and the allocation of nursing staff. As might be expected, shorter and less complex scales are more readily acceptable to busy staff members [13] and are also more convenient for patients.

### Methodological considerations

We employed the LASSO, which is a relatively new method for variable selection. In general, the task of selecting predictors in a sample containing more variables than observations poses serious problems if there is no a priori hypothesis, and if conventional regression analysis is employed. In addition, the analysis must deal with the problem of collinearity of the independent variables, i.e. several intercorrelated variables will produce inflated variances, and consequently unstable estimates. LASSO largely avoids the collinearity problem [48], and it is now generally accepted that LASSO yields more robust results than are afforded by classical variable selection methods such as stepwise forward or backward selection [49,50]. We find LASSO to have been useful for restriction of the ICF to operationally defined Core Sets and to have high predictive value for nursing workload.

### Limitations

Among the limitations of the present study, it must be conceded that the high face validity of the presented results from the obvious association between limitations in patients' activities, especially in self-care tasks, and the requirement for additional nursing interventions and modifications in treatment procedures. Since duties performed by nurses may vary between health care systems, some caution should be applied in generalizing conclusions with respect to country-specific responsibilities of nurses. Another limitation is related to the relatively small sample size per analysed group, which might have led to the exclusion of potentially important ICF categories in the models. However, the ideal number of independent variables was determined by cross-validating LASSO models, such that the final models with their included independent variables were validated on independent data sets, and proved to be superior to the other candidate models. As such, the number of variables was optimal under the given circumstances and given the available sample size. Another limitation might result from the large number of missing values for the variable Barthel Index in the musculoskeletal sample, for which there were only 50 observations. This is indeed a problematic state of affairs, given that the LEP and Barthel Index were administered to the same patients at only one of the study centre (KFJS). This limitation may moderate the extent of generalisability of our comparison of the predictive properties of the ICF categories and BI scores. Since the legal situation in Austria, Switzerland and Germany allows but a small proportion of unlicensed staff in acute hospital nursing services, the KFJS is certainly comparable to the other two study centres with respect to nursing skills. In addition, clinical nursing education is basically comparable in the three countries [51]. Finally, it must be conceded that nursing workload as measured by the summation of LEP variables is not quite equivalent to the real amount of time spent for patient-related nursing care. There is, however, a substantial body of work in which it has properly been assumed that the sum of LEP variables is highly correlated to nursing workload, thus constituting an adequate estimate of the outcome of interest [52]. In addition, a recent study of Baumberger et al. shows a nearly equal level of daily nursing workload measured by LEP in patients in Swiss acute hospitals [53]. The present exploratory findings may need support from empirical evidence in further studies involving larger patients groups.

## Conclusions

A substantial fraction of the variation in patient-related nursing workload in patients with rehabilitation needs in the acute care situation can be predicted by a highly selected group of categories of the Acute International Classification of Functioning, Disability and Health Core Sets.

Incorporating ICF Core Set-based data in nursing management decisions, particularly staffing decisions, may be beneficial. Considering patients functioning based on specific categories of the ICF may be an option for guiding staffing decisions at the unit-level as well as estimating full-time equivalents more precisely at the hospital-level. The result of our study may be extended to identify the predictors relevant for all health professionals involved in acute patient care. As such, this study represents the first step towards establishing a general approach enabling the entire interdisciplinary team to plan the patient-specific workload in a common language.

## Competing interests

The authors declare that they have no competing interests.

## Authors' contributions

MM, EG and CB designed the study and supervised the data collection. RS, SL and MM analysed the data. All authors interpreted the results and contributed in drafting the manuscript. All authors read and approved the final manuscript.

## Pre-publication history

The pre-publication history for this paper can be accessed here:

http://www.biomedcentral.com/1472-6963/10/295/prepub
